# *N*-n-butyl Haloperidol Iodide Protects against Hypoxia/Reoxygenation Injury in Cardiac Microvascular Endothelial Cells by Regulating the ROS/MAPK/Egr-1 Pathway

**DOI:** 10.3389/fphar.2016.00520

**Published:** 2017-01-05

**Authors:** Shishi Lu, Yanmei Zhang, Shuping Zhong, Fenfei Gao, Yicun Chen, Weiqiu Li, Fuchun Zheng, Ganggang Shi

**Affiliations:** ^1^Department of Pharmacy, the First Affiliated Hospital, Shantou University Medical CollegeShantou, China; ^2^Department of Pharmacology, Shantou University Medical CollegeShantou, China; ^3^Department of Biochemistry and Molecular Biology, University of Southern CaliforniaLos Angeles, CA, USA; ^4^Analytical Cytology Laboratory, Shantou University Medical CollegeShantou, China; ^5^Department of Clinical Pharmacology Laboratory, the First Affiliated Hospital, Shantou University Medical CollegeShantou, China; ^6^Department of Cardiovascular Diseases, the First Affiliated Hospital, Shantou University Medical CollegeShantou, China

**Keywords:** *N*-n-butyl haloperidol, reactive oxygen species, Egr-1, hypoxia/reoxygenation, cardiac microvascular endothelial cell, mitogen-activated protein kinase

## Abstract

Endothelium dysfunction induced by reactive oxygen species (ROS) is an important initial event at the onset of myocardial ischemia/reperfusion in which the Egr-1 transcription factor often serves as a master switch for various damage pathways following reperfusion injury. We hypothesized that an intracellular ROS/MAPK/Egr-1 signaling pathway is activated in cardiac microvascular endothelial cells (CMECs) following hypoxia/reoxygenation (H/R). ROS generation, by either H/R or the ROS donor xanthine oxidase-hypoxanthine (XO/HX) activated all three MAPKs (ERK1/2, JNK, p38), and induced Egr-1 expression and Egr-1 DNA-binding activity in CMECs, whereas ROS scavengers (EDA and NAC) had the opposite effect following H/R. Inhibitors of all three MAPKs individually inhibited induction of Egr-1 expression by H/R in CMECs. Moreover, *N*-n-butyl haloperidol (F_2_), previously shown to protect cardiomyocytes subjected to I/R, dose-dependently downregulated H/R-induced ROS generation, MAPK activation, and Egr-1 expression and activity in CMECs, whereas XO/HX and MAPK activators (EGF, anisomycin) antagonized the effects of F_2_. Inhibition of the ROS/MAPK/Egr-1 signaling pathway, by either F_2_, NAC, or inhibition of MAPK, increased CMEC viability and the GSH/GSSG ratio, and decreased Egr-1 nuclear translocation. These results show that the ROS/MAPK/Egr-1 signaling pathway mediates H/R injury in CMECs, and F_2_ blocks this pathway to protect against H/R injury and further alleviate myocardial I/R injury.

## Introduction

Reactive oxygen species (ROS), including hydrogen peroxide (H_2_O_2_), superoxide anion (${\text{O}}_{2}^{•-}$) and hydroxyl radical (·OH), are forms of oxygen free radicals that arise as by-products of mitochondrial respiration and oxidases. There is growing evidence that ROS generated from excess oxidative stress are responsible for many cardiovascular diseases, including hypertension, diabetes, and ischemia/reperfusion (I/R)-related heart diseases (Fennell et al., 2002; Jung et al., 2003; Yang et al., 2004; Liu et al., 2013). Restoration of blood in ischemic organs induces further injury, called I/R injury, by a mechanism involving ROS-induced oxidative stress (Murphy and Steenbergen, 2008). Upon I/R, excessive accumulation of ROS can disrupt cellular homeostasis, resulting in oxidative stress damage and additional I/R injury. As a second messenger (Cosentino-Gomes et al., 2012; Choudhury et al., 2013; Madureira and Waisman, 2013), ROS not only cause direct damage to cellular proteins, lipids and nucleic acids, but also are capable of acting as signaling molecules to initiate damage or survival signals. In recent years, research focusing on I/R suggests that ROS are largely responsible for I/R injury (Zhang et al., 2012; He et al., 2013; Wang et al., 2014).

Early growth response gene-1 (Egr-1) is an immediate early gene that functions extensively in cellular growth, proliferation, differentiation, and apoptosis. It has been reported that Egr-1 can be rapidly induced by various stimuli, including growth factors, ischemia, hypoxia, and oxidative stress, to regulate many pathological progresses. As a transcription factor, Egr-1 can transmit information from the cytoplasm to nucleus, and then alter downstream target gene expression to mediate I/R injury. In 2000, Yan et al. postulated that Egr-1 is a “master switch” for various pathways of reperfusion injury (Yan et al., 2000), and since then, Egr-1 has been investigated extensively due to its key role in I/R.

Recent research suggests that there exists a connection between ROS and Egr-1. Han et al. (2013) and Kang et al. (2013) reported that the anticancer actions of sanguinarine and 2′-benzoyloxycinnamaldehyde are related to activation of the ROS/Egr-1 pathway. A study by Nozik-Grayck et al. (2008) revealed that hypoxia-induced overexpression of Egr-1 is inhibited in transgenic mice overexpressing extracellular superoxide dismutase (EC-SOD) to eliminate ROS. In addition, a new study by our laboratory found that hypoxia/reoxygenation (H/R, I/R model *in vitro*) activates the ROS/Egr-1 pathway in cardiac-derived H9c2 rat cardiomyoblast cells (Zhang et al., 2015). However, cardiac microvascular endothelial cells (CMECs), a major component of myocardial tissue, are the earliest components of the heart exposed to I/R stimulation and are quite sensitive to I/R injury (Brutsaert, 2003). Previous studies on CMECs, as well as H9c2 cells, revealed that oxidative stress and Egr-1 expression contribute to H/R injury (Zhou et al., 2010b). Moreover, damage resulting from oxidative stress decreases in the presence of Egr-1 antisense oligonucleotide, indicating that the ROS/Egr-1 pathway might exist in CMECs and be responsible for H/R injury in CMECs and even heart tissue (Zhou et al., 2010a). However, a ROS/Egr-1 pathway in CMECs has yet to be identified.

The mitogen-activated protein kinase (MAPK) family, which includes the extracellular signal-regulated kinases (ERK1/2), stress-activated protein kinase/c-Jun N-terminal kinase (SAPK/JNK), and p38 MAP kinase (p38 MAPK), is evolutionarily conserved and known to respond to stress through transmission of activating signals along a phosphorylation cascade from cytoplasmic to nuclear targets. Extensive studies demonstrate that MAPKs (ERK1/2, JNK, p38) are involved in I/R injury. We previously demonstrated that JNK and ERK1/2 mediate Egr-1 induction during H/R injury in H9c2 cells and primary cardiomyocytes, respectively, with ROS/Egr-1 signaling participating in the former (Zhang et al., 2013, 2015). It remains unknown whether MAPK is activated by H/R stimuli in CMECs, and whether the other two MAPK pathways, besides JNK, are involved in CMEC ROS/Egr-1 signaling.

*N*-n-butyl haloperidol iodide (F_2_) is a novel compound, derived from haloperidol and granted by China (Supplementary Images 1, 2), that has a protective effect on heart tissue subjected to I/R. F_2_ could protect against H/R injury in CMECs, as well as H9c2 cells, by decreasing oxidative stress and inhibiting the expression of Egr-1 (Zhou et al., 2010b). Furthermore, F_2_ alleviates H/R injury by reducing the activation of JNK and ERK1/2 responsible for Egr-1 induction in H9c2 cells and primary cardiomyocytes following H/R (Zhou et al., 2010b; Zhang et al., 2013). The above data suggest that the protective effect of F_2_ on CMECs could be related to an ability to inhibit the ROS/MAPK/Egr-1 pathway. This study was implemented to determine whether the ROS/MAPK/Egr-1 signaling pathway is active in CMECs subjected to H/R, and if the protective effect of F_2_ on CMECs and heart tissue results from the ability of F_2_ to inhibit this pathway.

## Materials and methods

### Reagent preparation

Fetal bovine serum (FBS) and Dulbecco's modified Eagle's medium (DMEM) were obtained from Gibco. Endothelial cell growth supplement (ECGS) was from Merck Millipore. F_2_ (Chinese national invention patent, No. ZL96119098.1) was synthesized in our laboratory and used at concentrations of 1 × 10^−7^, 1 × 10^−6^, 1 × 10^−5^ M (DMSO as solvent). Edaravone (EDA) was from Simcere Pharmaceuticals (Nanjing, China). N-acetyl-L-cysteine (NAC), xanthine oxidase (XO)/hypoxanthine (HX), anisomycin (ANISO), U0126, SB203580 and 2′,7′-dichlorofluorescein acetyl acetate (DCFH-DA, mainly detecting H_2_O_2_) were from Sigma-Aldrich. SP600125 was from Enzo Life Sciences. Epidermal growth factor (EGF) was from Pepro Tech.

Primary antibodies against p-JNK, total-JNK, p-ERK1/2, total-ERK1/2, p-p38, total-p38, and Egr-1 were purchased from Cell Signaling Technology (United States); primary antibody against platelet endothelial cell adhesion molecule-1 (CD31) was purchased from Bio-RAD (United States). Antibodies against β-actin, anti-rabbit secondary antibody, and anti-mouse secondary antibody were purchased from Wuhan Boster Biotechnology Limited (Wuhan, China). The EMSA assay kit was purchased from Thermo-Fisher Scientific (United States).

Hypoxia solution (Zhang et al., 2015): 137 mM NaCl, 12 mM KCl, 0.49 mM MgCl_2_·6H_2_O, 0.9 mM CaCl_2_, 4 mM HEPES, and 20 mM sodium lactate. Binding buffer for EMSA: HEPES pH 7.5, 5 mM MgCl_2_, 2.5 mM dithiothreitol, 2.5 mM EDTA, 250 mM NaCl, and 10% glycerol.

### Primary cell isolation culture and induction of hypoxia/reoxygenation

Neonatal Sprague-Dawley (SD, 3–5 days) rats of either sex were used in experiments. All animals were treated in compliance with the Guide for Care and Use of Laboratory Animals published by the US National Institutes of Health (NIH publication No. 85-23, revised 1996) and followed the rules of National Animal Protection of China. The study was approved by the Institutional Animal Care and Use Committee of Shantou University Medical College. Rat CMECs were isolated as described (Zhou et al., 2010a) and cultured in DMEM supplemented with 10% FBS, ECGS (15 mg/L) and heparin sodium (6.25 U/ml) at 37°C under 5% CO_2_. CMECs were identified by antibody against CD31, which is constitutively expressed on the surface of CMECs. Hypoxia was induced as before with some modifications (Zhou et al., 2010a). Briefly, CMECs were cultured in hypoxia solution in an air-tight chamber saturated with pure N_2_ at 37°C for 1, 2, 3, or 4 h; the culture conditions were then returned to normal for 1 h of reoxygenation.

### Experimental grouping

**Figure 1:**
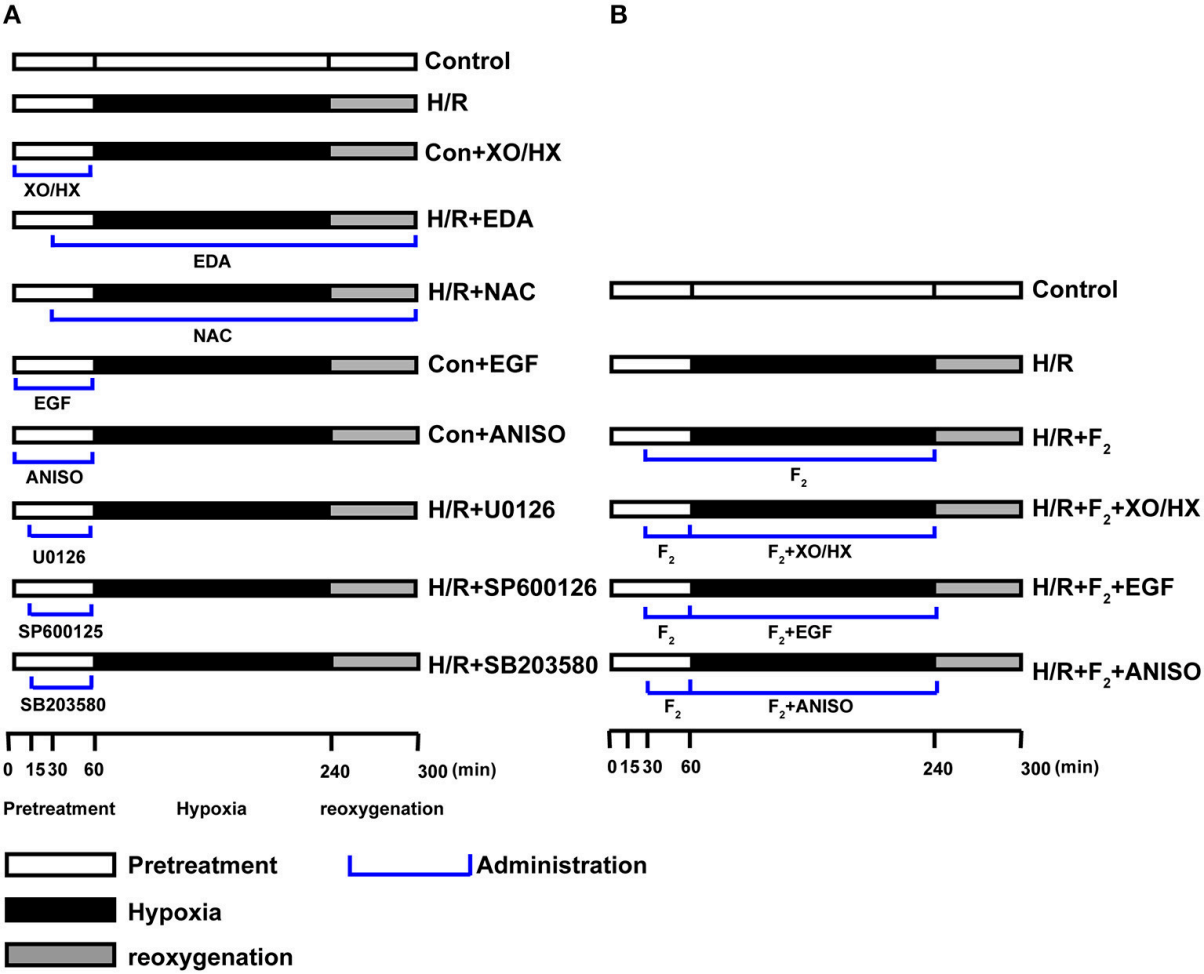
**Protocol for experimental grouping and treatments**. **(A)** Protocol used to investigate whether the ROS/MAPK/Egr-1 signaling pathway occurs in CMECs after H/R. **(B)** Protocol used to investigate the protective effects of F_2_ on CMEC H/R injury through regulating ROS/MAPK/Egr-1 signaling.

CMECs after being cultured for 2–3 days were randomly allocated to the following groups: control, control + the ROS donor XO/HX (con + XO/HX), control + the ERK1/2 activator EGF (con + EGF), control + the JNK/p38 activator ANISO (con + ANISO), H/R, H/R + the ROS scavenger NAC (H/R + NAC), H/R + the ROS scavenger EDA (H/R + EDA), H/R + ERK1/2 inhibitor U0126 (H/R + U0126), H/R + JNK inhibitor SP600125 (H/R + SP600125), H/R + p38 inhibitor SB203580 (H/R + SB203580), H/R + different doses of F_2_, H/R + F_2_ + XO/HX, H/R + F_2_ + EGF, and H/R + F_2_ + ANISO. The working concentrations of each chemical and solvent used to prepare stock solutions are as follows: XO (1, 3, 5 mU/ml, potassium phosphate buffer as solvent)/HX (1.2 × 10^−4^, 3.6 × 10^−4^, 6.0 × 10^−4^, 10 M NaOH as solvent), EGF (50 ng/ml, H_2_O as solvent), ANISO (40 ng/ml, DMSO as solvent), NAC (2 × 10^−5^, 1 × 10^−4^, 5 × 10^−4^ M, H_2_O as solvent), EDA (5 × 10^−5^, 1 × 10^−4^, 2 × 10^−4^ M, PEG as solvent), U0126 (10 μM, DMSO as solvent), SP600125 (10 μM, DMSO as solvent), SB203580 (20 μM, DMSO as solvent), F_2_(1 × 10^−7^, 1 × 10^−6^, 1 × 10^−5^ M DMSO as solvent). CMECs in all groups were cultured in serum-free medium for 12–24 h to achieve quiescence. After that, cells in the control group were left intact. The H/R group was treated as described above. The concentration and administration protocol for XO/HX, EGF, ANISO, NAC, EDA, F_2_, SP600125, U0126, and SB203580 were described in “Reagent preparation” and Figure 1, respectively.

### Measurement of ROS levels in CMECs by using flow cytometry

Flow cytometry was performed as previously described (Zhang et al., 2015). In short, cells were harvested after the indicated treatments, washed twice with PBS, and then washed once with serum-free medium. Subsequently, the supernatants were discarded and cell pellets were resuspended in 1 ml serum-free medium containing a final concentration of 5 μM DCFH-DA, and then incubated in the dark at 37°C for 30 min with gentle shaking every 5 min to fully expose the cells to the probe. Cell suspensions were then washed three times with ice-cold PBS. Subsequently, the cell pellets were resuspended in 500 μl PBS, mixed well, and analyzed at an excitation wavelength of 488 nm and emission wavelength of 525 nm using a FACSCalibur flow cytometer (Becton Dickinson, USA). WinMDI2.9 software was used to analyze the mean fluorescence intensity (MFI).

### Western blotting

Western blotting was performed as described previously (Zhang et al., 2015). Briefly, total protein in CMECs was extracted and quantified. Next, equal amounts of denatured protein samples (20–50 μg) were subjected to 8% SDS-PAGE and probed with corresponding primary antibodies for Egr-1 (1:1500), p-JNK (1:1000), total-JNK (1:2000), p-ERK1/2 (1:2000), total-ERK1/2 (1:2000), p-p38 (1:2000), total-p38 (1:2000), and β-actin (1:4000) at 4°C overnight, followed by secondary antibodies [HRP-labeled rabbit anti-mouse IgG (1:50,000) or HRP-labeled goat anti-rabbit IgG (1:80,000)]. HRP-labeled secondary antibodies were detected by chemiluminescence, and the protein bands were analyzed using Gel-pro Image Analysis Software (Media Cybernetics, USA). The ratio of p-JNK/JNK, p-ERK1/2/ERK1/2, and p-p38/p38 reflected the activation of JNK, ERK1/2, and p38, respectively, and the ratio of Egr-1/β-actin represented the expression of Egr-1.

### RNA extraction and real-time quantitative PCR

The expression level of Egr-1 mRNA was detected by qRT-PCR. Total RNA was extracted from CMECs using the RNAiso Plus kit (Takara). Total RNA (0.5 μg) was used to synthesize cDNA by using a PrimeScript RT Reagent Kit with gDNA Eraser (Takara) according to the manufacture's protocol. CDNAs were then quantified by RT-PCR on an ABI 7500 RT-PCR System (Applied Biosystems) using the following primers, Egr-1: 5′-GAACAACCCTACGAGCACCTG-3′ (sense), 5′-GCCACAAAGTGTTGCCACTG-3′ (antisense); GAPDH: 5′-GGCACAGTCAAGGCTGAGAATG-3′ (sense), 5′-ATGGTGGTGAAGACGCCAGTA-3′ (antisense), which were synthesized by BGI (BGI, China). GAPDH was used as an endogenous reference and the ratio of Egr-1 mRNA/GAPDH mRNA represented expression of the Egr-1 gene.

### Immunofluorescence detection of CD31 and Egr-1

CMECs were inoculated at 1.0 × 10^−5^ cells/well on coverslips in 12-well-plates. When cells grew to 70% confluence, they were treated with the conditions as indicated. Subsequently, cells were fixed in 4% paraformaldehyde for 20 min at room temperature, permeabilized in 0.3% Triton X-100 for 15 min, and then were blocked in 5% BSA for 1 h. Next, BSA was removed and cells were incubated, with primary antibodies recognizing CD31 (1:50) and Egr-1 (1:50), at 4°C overnight, followed by AlexaFlour 488-conjugated goat anti-mouse or AlexaFlour 594-conjugated goat anti-rabbit secondary antibodies for 1–2 h at room temperature. Subsequently, the cells were washed in PBS and counterstained with Hoechst 33258. Then, coverslips were mounted onto glass slides using fluorescence mounting medium (Beyotime Biotechnology), and the fluorescence signal was detected using 200 × magnification on a Nikon microscope. Exposure times for each channel were as follows: Hoechst 33258, 200 ms; AlexaFlour 488, 200 ms; AlexaFlour 594, 600 ms.

### Detecting DNA binding activity of Egr-1 using EMSA

Electrophoretic mobility shift analysis (EMSA) was performed on nuclear extracts prepared from CMECs. Complementary 27-bp oligonucleotides containing an Egr-1 binding site were 5′-GGATCCAGCGGGGGCGAGCGGGGGCCA-3′, 5′-TGGCCCCCGCTCGCCCCCGCTGGATCC-3′. Oligonucleotides were 5′-end-labeled with biotin and annealed. The biotin-labeled probe was incubated with 6 μg protein and poly (dI-dC) (50 μg/ml) in binding buffer for 25 min at room temperature. Gels were pre-run for 45 min, then samples were loaded directly onto non-denaturing polyacrylamide/bisacrylamide (6%) gels. Electrophoresis for 1.5–2 h at 100 volts, and electrotransfer for 45 min at 380 mA were performed in an ice bath. For competition studies, 50- and 200-fold molar excesses of unlabeled probe for Egr-1 were added.

### Detecting GSH/GSSG ratio and MDA level

The intracellular glutathione/oxidized glutathione (GSH/GSSG) ratio, which reflects the oxidative stress level and the ability to eliminate ROS, was measured with the GSH and GSSG Assay Kit (Beyotime Technology, China). Briefly, cells were harvested after the indicated treatments and were mixed with protein removal reagent (3 times the cell volume, 10 mg ≈ 10 μl). Subsequently, cells were freeze-thawed two times in liquid nitrogen and 37°C water. Cells were placed in an ice bath for 10 min, and then centrifuged for 10 min at 4°C and 10,000 × g, after which the supernatant was assayed for total GSH and GSSG according to the manufacturer's instructions. Absorbance was measured at 412 nm over 25 min. The concentration of reduced GSH in the sample was obtained by subtracting GSSG from total-GSH.

Malonaldehyde (MDA) was measured by thiobarbituric acid (TBA) reactivity using an MDA assay kit (Nanjing Jiancheng Bioengineering Institute, China) according to the manufacture's protocol. Supernatants containing MDA were obtained from the cytoplasm of CMECs. The concentration of MDA was calculated by a calibration curve using 1,1,3,3′tetra-ethoxy propane as a standard.

### Assessment of CMEC viability by MTT assay

CMECs were inoculated in 96-well-plates. After different treatments with NAC, F_2_, U0126, SP600125, and SB203580, 3-[4, 5-dimethylthiazol-2-yl]-2, 5 diphenyltetrazolium bromide (MTT) solution was added to the medium and cells were incubated for an additional 4 h at 37°C. Then the medium was discarded and DMSO was added to dissolve the formazan crystals. All operations were performed in the dark. Absorbance was measured at 490 nm using a SpectraMax M2e Microplate Reader (Molecular Devices). The control group was considered as 100% viable.

### Statistical analysis

All data are presented as mean ± S.E.M. Differences between groups were determined using one-way ANOVA followed by a Student-Newman-Keuls test with SPSS 17.0 software. Statistical significance was considered at a value of *P* < 0.05.

## Results

### Derivation of CMECs

Microvascular endothelial cells isolated from heart of neonatal Sprague-Dawley rats were multipolar and possessed a cobblestone-like appearance upon reaching confluence. CD31 is constitutively expressed on the surface of CMECs. As judged by immunofluorescence, all cells were CD31-positive, confirming that the cells were CMECs (Supplementary Image 3).

### ROS level and Egr-1 protein expression in CMECs at various times following H/R

**Figure 2:**
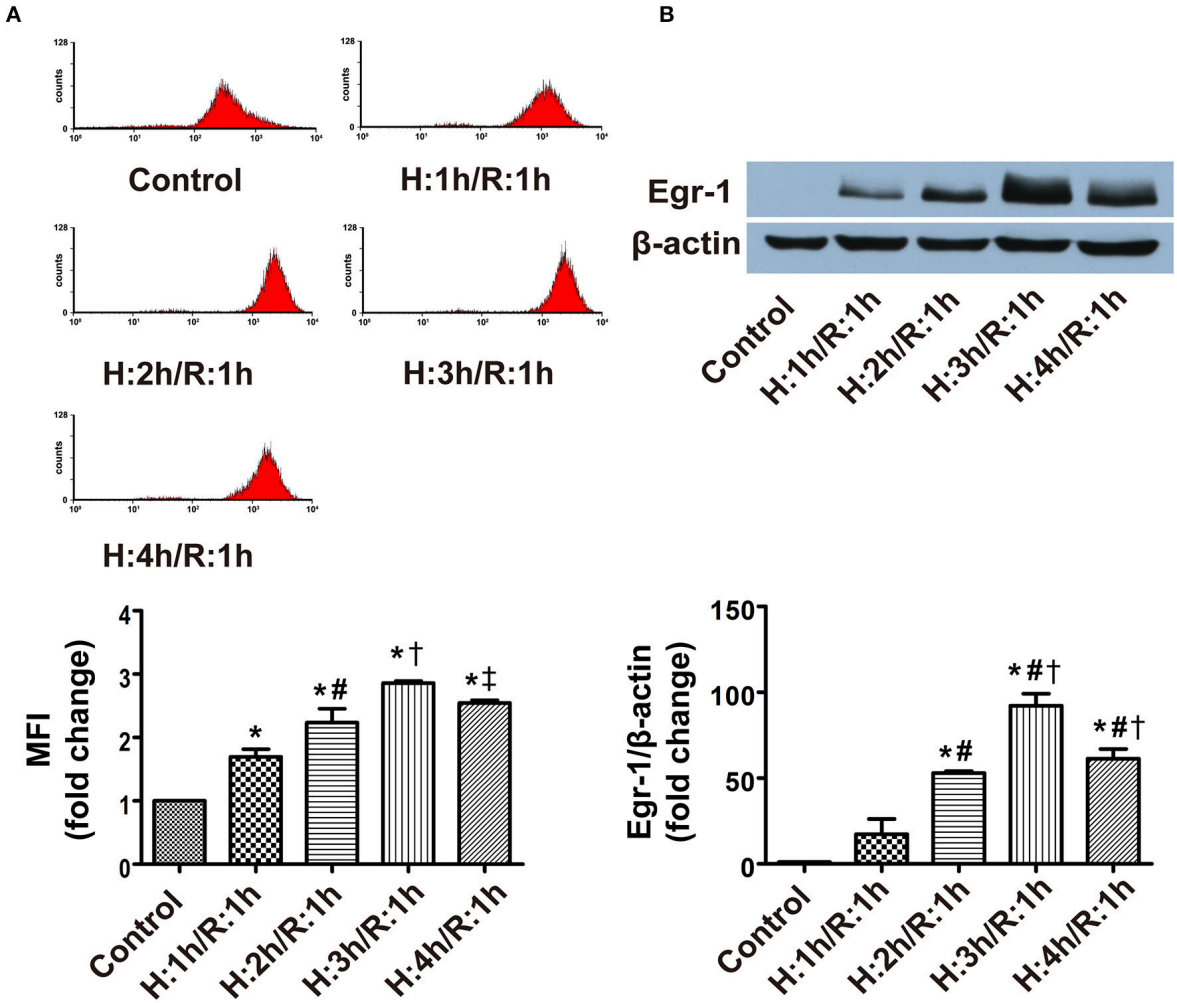
**ROS level and Egr-1 protein expression in CMECs following different durations of hypoxia, and a 1-h reperfusion, as assessed using flow cytometry and western blotting. (A)** ROS levels during H/R; *n* = 3. **(B)** Protein levels of Egr-1 and β-actin; *n* = 3. Quantitative data are expressed as the percentages of the control group. All values are expressed as mean ± S.E.M.^*^P < 0.05 vs. control; ^#^*P* < 0.05 vs. the H1/R1 group; ^†^*P* < 0.05 vs. the H2/R1 group; ^‡^*P* < 0.05 vs. the H3/R1 group.

To determine the effects of different durations of H/R on ROS level and Egr-1 expression, CMECs were cultured in an air-tight chamber saturated with pure N_2_ at 37°C for 1, 2, 3, or 4 h, then returned to normal conditions for 1 h of reoxygenation. Flow cytometry analysis revealed that ROS levels in CMECs increased in a manner dependent on the duration of H/R (≈1.7–2.9 fold), with peak ROS levels occurring at 3 h of hypoxia and 1 h of reoxygenation (H3/R1) vs. normoxia (Figure 2A). Immunoblotting of extracts prepared from CMECs showed expression of Egr-1 increased in all H/R groups (≈17–92-fold) compared with the control group. Concomitant with the peak in ROS levels, peak Egr-1 expression also occurred at H3/R1 (Figure 2B), followed by a decline in ROS generation and Egr-1 protein expression by H4/R1. Based on the above data, all subsequent experiments were performed using 3 h of hypoxia and 1 h of reoxygenation.

### Relationship between ROS level and Egr-1 expression during H/R

The correlation of time-dependent changes of Egr-1 protein, during H/R, with ROS levels suggested that ROS could be responsible for Egr-1 induction. To further investigate the cause-effect relationship between ROS and Egr-1 in H/R CMECs, we determined whether the ROS donor XO/HX and ROS scavengers EDA and NAC could exert changes in Egr-1 expression.

**Figure 3:**
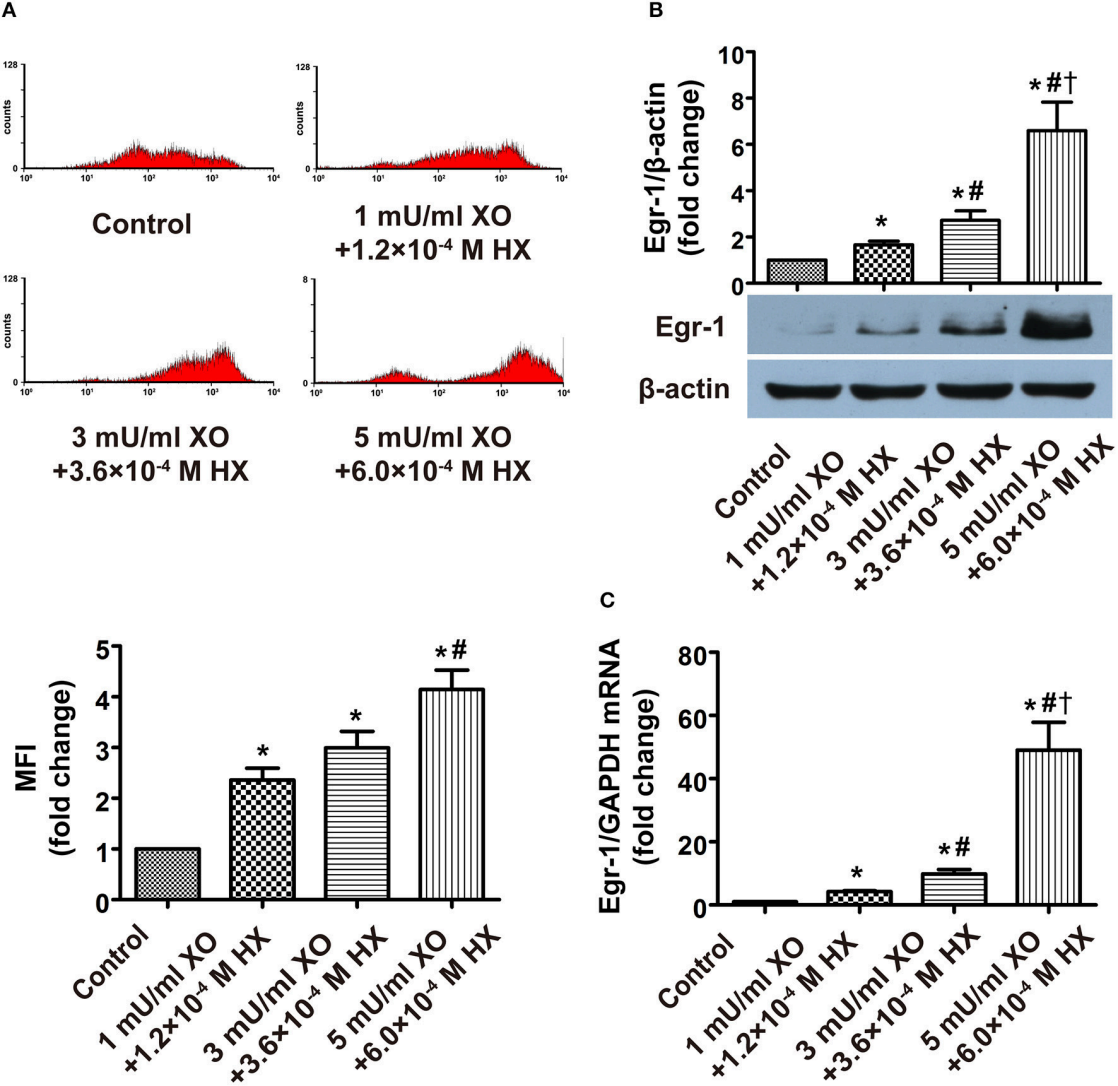
**Effects of different doses of a ROS donor on ROS level, and Egr-1 gene and protein expression in CMECs. (A)** Flow cytometry was performed to determine ROS levels; *n* = 4. **(B)** Egr-1 protein expression was detected by western blot; *n* = 3. **(C)** RT-PCR was performed to determine Egr-1 mRNA levels; *n* = 3. Quantitative data are expressed as the percentages of the control group. All values are expressed as mean ± S.E.M. ^*^P < 0.05 vs. control; ^#^*P* < 0.05 vs. the 1 mU/ml XO + 1.2 × 10^−4^ M HX group; ^†^*P* < 0.05 vs. the 3 mU/ml XO + 3.6 × 10^−4^ M HX group.

**Figure 4:**
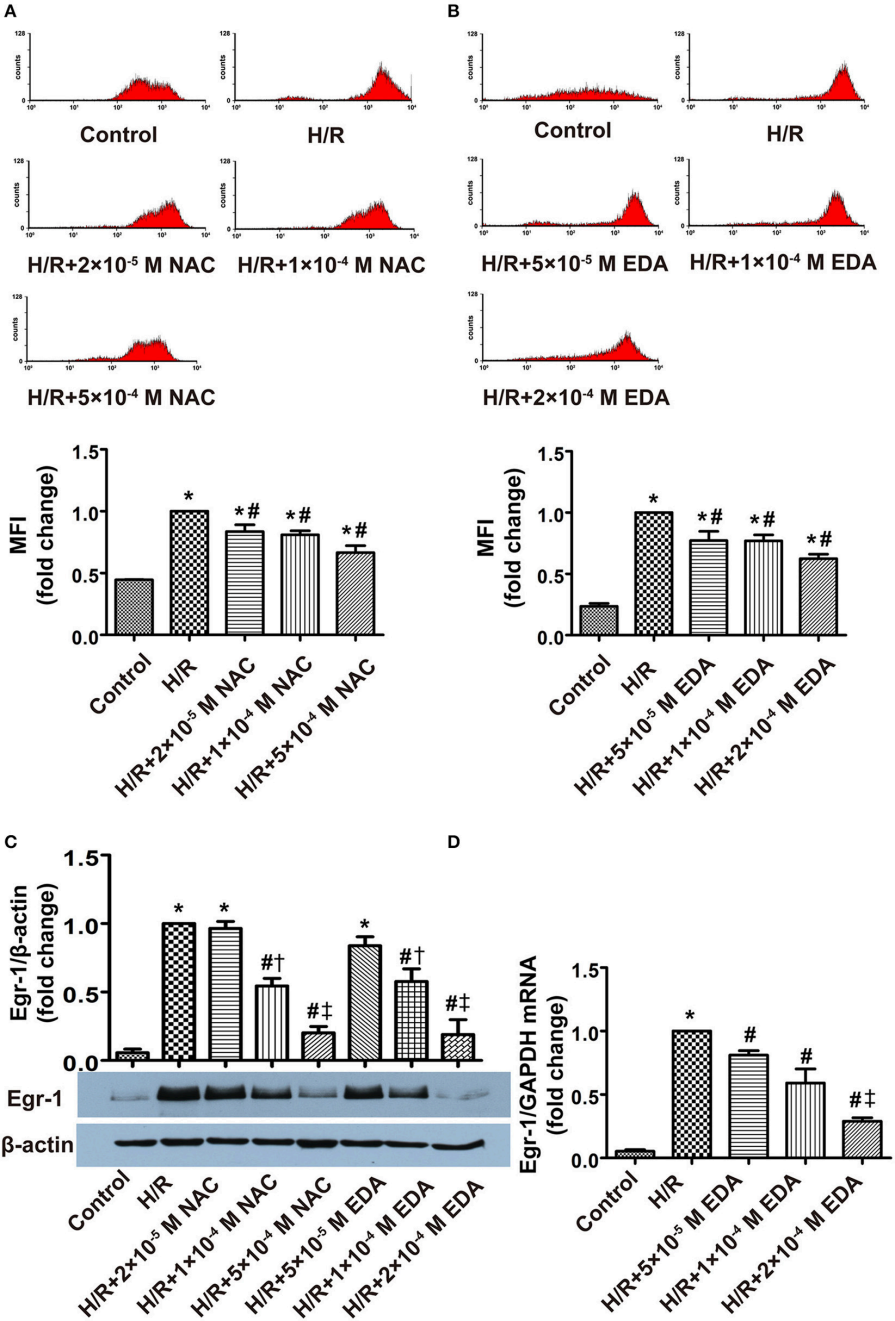
**Effects of different doses of ROS scavengers on ROS level, and Egr-1 gene and protein expression in CMECs after H/R, as assessed by flow cytometry, western blot and RT-PCR. (A)** Effect of NAC on ROS levels in H/R CMECs; *n* = 3. **(B)** Effect of EDA on ROS levels in H/R CMECs; *n* = 3. **(C)** Effects of EDA and NAC on protein levels of Egr-1 and β-actin; *n* = 3. **(D)** Effect of EDA on Egr-1 mRNA levels; *n* = 3. Quantitative data are expressed as the percentages of H/R groups. All values are expressed as mean ± S.E.M. ^*^P < 0.05 vs. control; ^#^*P* < 0.05 vs. H/R; ^†^*P* < 0.05 vs. H/R + 2 × 10^−5^ M NAC or H/R + 5 × 10^−5^ M EDA; ^‡^*P* < 0.05 vs. H/R + 1 × 1 0^−4^ M NAC or H/R + 1 × 10^−4^ M EDA.

Flow cytometric analysis of ROS, western blot analysis of Egr-1 protein and RT-PCR analysis of Egr-1 RNA harvested from XO/HX-induced CMECs displayed increased ROS levels, Egr-1 protein expression and Egr-1 transcripts (≈2.4-, 1.7-, 4.2-fold, respectively) at low concentrations of XO/HX, which rose further at moderate concentration of XO/HX (≈3.0-, 2.7-, 9.8-fold, respectively), and were even more elevated at high concentration of XO/HX (≈4.1-, 6.6-, 49-fold, respectively), as compared with the control group (Figures 3A–C), demonstrating that treatment with different concentrations of XO/HX for 1 h increased ROS levels and Egr-1 mRNA and protein expression in a dose-dependent manner. Pretreatment with EDA and NAC for 30 min decreased ROS levels and Egr-1 mRNA expression induced by H/R (Figures 4A,B,D), with moderate and high concentrations of ROS scavengers (1 × 10^−4^ M and 2 × 10^−4^ M EDA, 1 × 10^−4^ and 5 × 10^−4^ M NAC) resulting in substantial decreases in H/R-induced Egr-1 protein expression (Figure 4C). In contrast, low concentrations of ROS scavengers (5 × 10^−5^ M EDA, 2 × 10^−5^ M NAC) had no significant effect on Egr-1 expression induced by H/R. These results indicate that Egr-1 expression in CMECs is regulated by ROS levels following H/R stimulation. In other words, a ROS/Egr-1 signaling pathway is activated in CMECs by H/R.

### MAPKs mediate ROS/Egr-1 signal transduction in H/R CMECs

**Figure 5:**
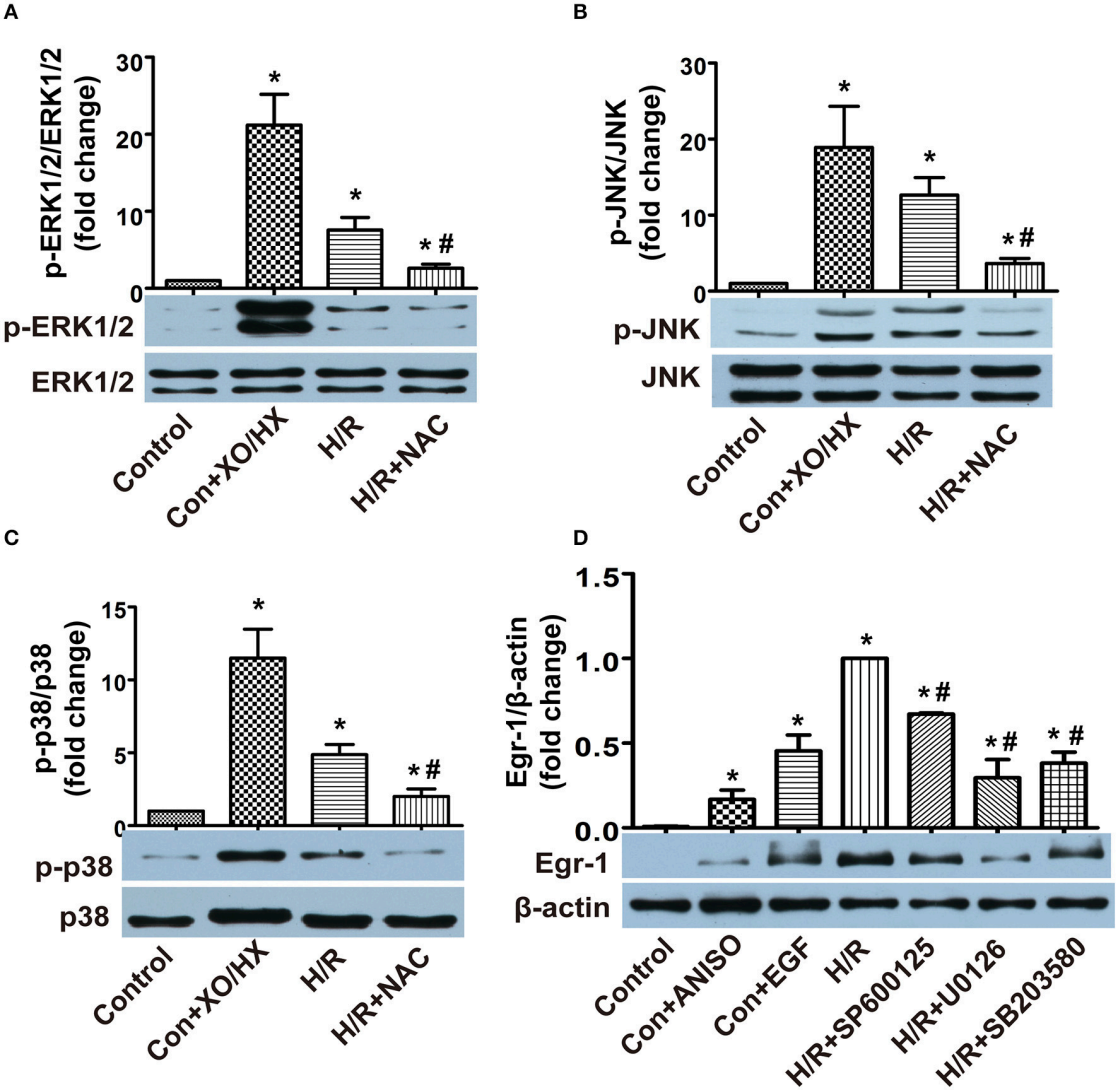
**Effects of MAPKs (ERK1/2, JNK, and p38) on ROS/Egr-1 signaling in CMECs subjected to H/R, as detected by western blotting**. **(A)** Effects of XO/HX and NAC on ERK1/2; *n* = 3. **(B)** Effects of XO/HX and NAC on JNK; *n* = 4. **(C)** Effects of XO/HX and NAC on p38; *n* = 3. **(D)** Effects of MAPK activators and inhibitors on expression of Egr-1 protein. *n* = 3. Quantitative densitometric data are expressed as percentages of the control or H/R groups. All values are presented as mean ± S.E.M.^*^P < 0.05 vs. control; ^#^*P* < 0.05 vs. H/R.

To observe the relationship between ROS and MAPK activity, we initially used XO/HX (5 mU/ml, 6.0 × 10^−4^ M) to increase ROS levels, and NAC (5 × 10^−4^ M) to decrease H/R-induced ROS generation, and examined MAPK activity. We found that p-ERK1/2, p-JNK, and p-p38 protein expression all increased in XO/HX-treated CMECs (≈21-, 19-, 11-fold, respectively) and H/R-induced CMECs (≈8-, 13-, 5-fold, respectively) compared with the control CMECs, indicating ERK1/2, JNK, and p38 were all activated by ROS and H/R (Figures 5A–C). H/R-mediated ERK1/2, JNK, and p38 activation was decreased significantly by addition of the ROS scavenger NAC (Figures 5A–C). We next utilized activators and inhibitors of MAPKs to assess if activation of MAPK by ROS regulates the expression of Egr-1. Addition of ANISO (40 ng/ml) for 1 h, to activate JNK/p38, or addition of EGF (50 ng/ml) for 1 h, to activate ERK1/2, both increased Egr-1 protein expression, whereas the H/R-induced increase in Egr-1 protein could be blocked by preincubation of cells with SP600125 (10 μM), U0126 (10 μM), or SB203580 (20 μM) for 45 min (Figure 5D). These data suggest that ERK1/2, JNK, and p38 are all involved in ROS/Egr-1 signal transduction, and that activation of these MAPKs plays an important role in the ROS/Egr-1 pathway.

### Effects of F_2_ on ROS level, MAPK activation, and Egr-1 expression and activity

**Figure 6:**
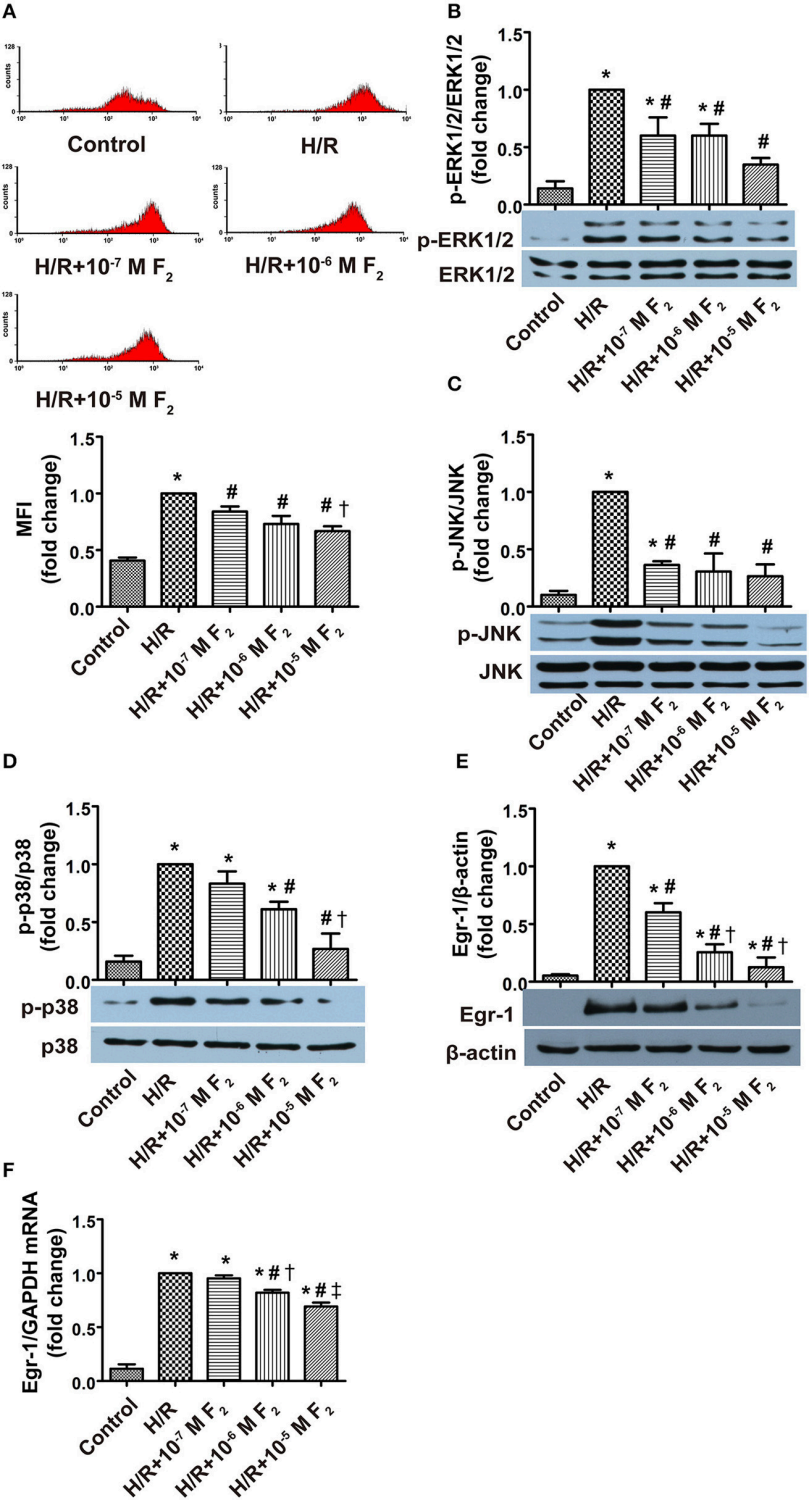
**Effects of F_**2**_ on ROS level, MAPK (ERK1/2, JNK, and p38) activation, and Egr-1 gene and protein expression in CMECs after H/R, as assessed using flow cytometry, western blotting and RT-PCR**. **(A)** ROS levels; *n* = 3. **(B)** Total ERK1/2 and p-ERK1/2 protein expression; *n* = 4. **(C)** Total JNK and p-JNK protein expression; *n* = 3. **(D)** Total p38 and p-p38 protein expression; *n* = 3. **(E)** Egr-1 and β-actin protein expression; *n* = 3. **(F)** Egr-1 mRNA and GAPDH mRNA expression; *n* = 3. Quantitative data are expressed as percentages of the levels of H/R groups. All values are expressed as mean ± S.E.M. ^*^P < 0.05 vs. control; ^#^*P* < 0.05 vs. H/R; ^†^*P* < 0.05 vs. H/R + 10^−7^ M F_2_; ^‡^*P* < 0.05 vs. H/R + 10^−6^ M F_2_.

**Figure 7:**
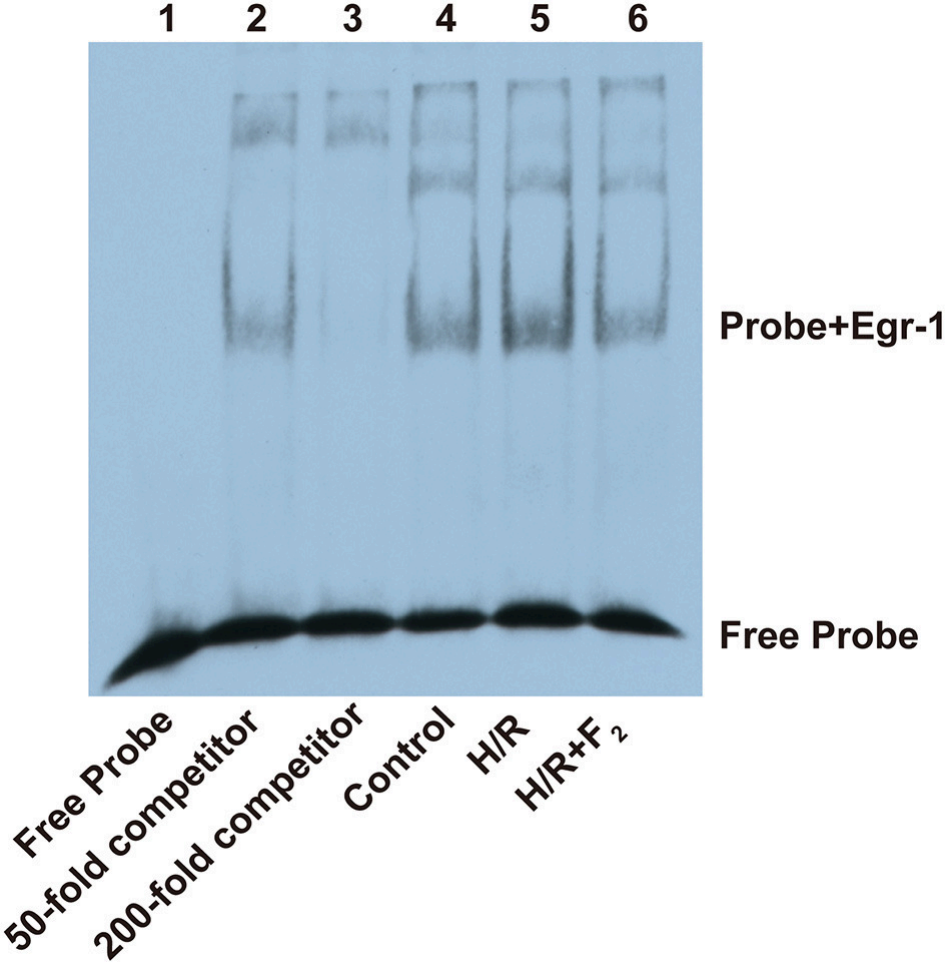
**Effect of F_**2**_ on DNA binding activity of Egr-1**. EMSA was performed with a biotin-labeled consensus Egr-1 oligonucleotide probe and CMEC nuclear extracts (6 μg/lane of nuclear protein). Competitor samples were incubated with an extra 50- or 200-fold non-biotin-labeled probe.

After confirming H/R-mediated activation of the ROS/MAPK/Egr-1 signaling pathway in CMECs, we next explored the effects of F_2_ on this pathway. Compared with the control group, ROS level, MAPK activation, and Egr-1 mRNA and protein expression all increased in the H/R group. However, these increases were dose-dependently inhibited by a 30 min pretreatment with 0.1–10 μM F_2_ (Figures 6A–F). High concentrations of F_2_ further decreased ROS levels compared with low concentrations of F_2_ (Figures 6A). Moderate and high F_2_ concentrations reduced Egr-1 protein expression to a greater extent compared with low concentrations (Figure 6E). In addition, moderate and high F_2_ concentrations decreased the expression of Egr-1 mRNA and p38 activation, as compared with low concentrations of F_2_ (Figures 6F,D). As evidenced by EMSA of nuclear extracts from CMECs, using a biotin-labeled oligonucleotide probe containing a consensus Egr-1 binding site, increased DNA binding activity of Egr-1 was observed after H/R, and the increase was inhibited by F_2_ pretreatment (Figure 7). These data suggest that F_2_ inhibits H/R-induced ROS, MAPK activation, Egr-1 expression, and enhanced Egr-1 DNA binding in CMECs in a dose-dependent manner. Although we have previously shown that inhibiting expression of Egr-1 is one of the mechanisms by which F_2_ protects cardiomyocytes from H/R injury (Zhang et al., 2007), we extend these results to show that F_2_ affects the DNA binding activity of Egr-1.

### ROS/MAPK/Egr-1 pathway agonists antagonize the effects of F_2_

**Figure 8:**
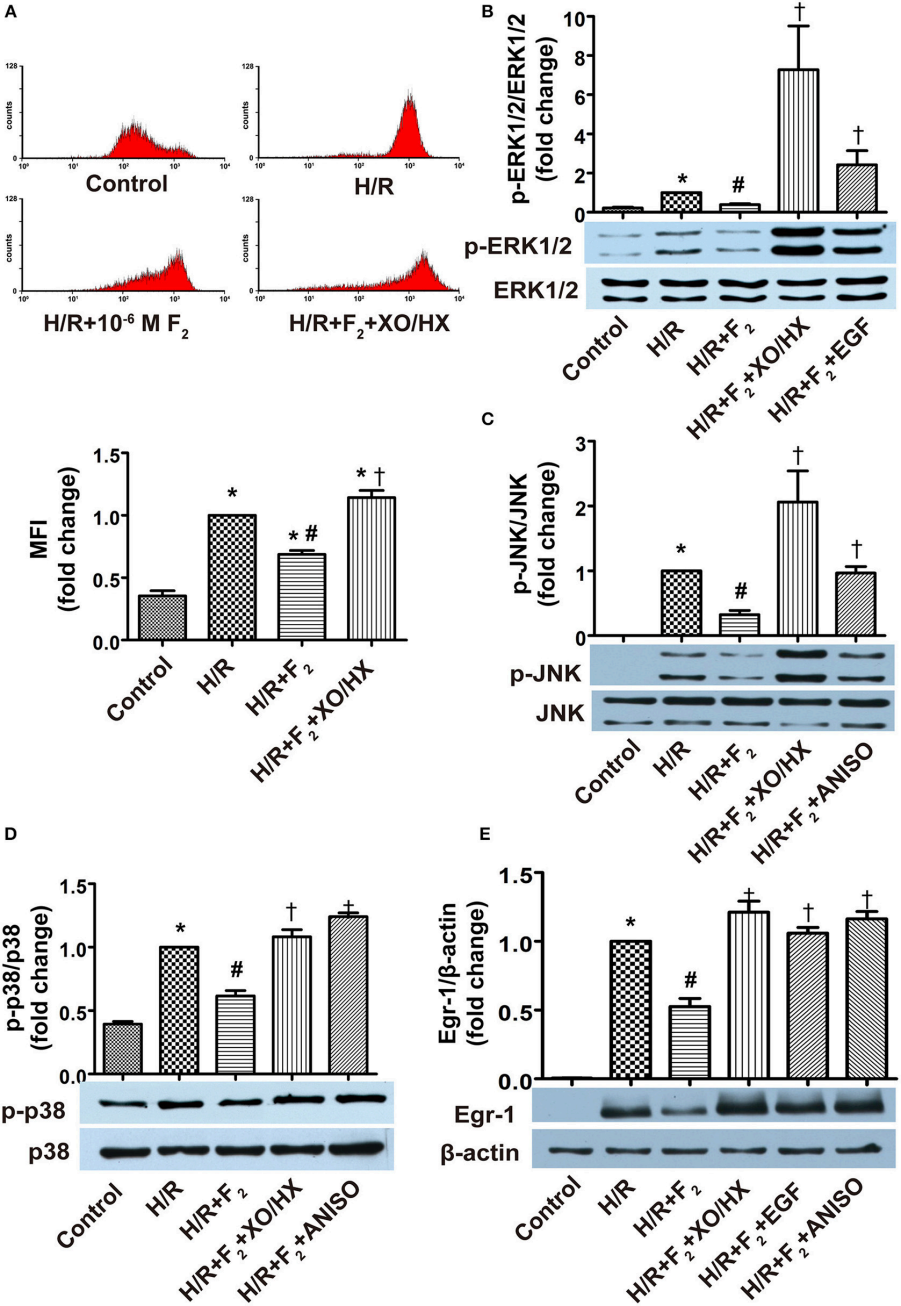
**Influence of ROS donor and MAPK activators on the effects of F_**2**_ on ROS level, MAPK activation, and Egr-1 expression in CMECs after H/R, as determined using flow cytometry and western blot. (A)** ROS levels; *n* = 3. **(B)** Total ERK1/2 and p-ERK1/2 protein expression; *n* = 3. **(C)** Total JNK and p-JNK protein expression; *n* = 3. **(D)** Total p38 and p-p38 protein expression; *n* = 3. **(E)** Egr-1 and β-actin protein expression; *n* = 3. Quantitative data are expressed as percentages of the levels of the H/R groups. All values are expressed as means ± S.E.M. ^*^P < 0.05 vs. control; ^#^*P* < 0.05 vs. H/R; ^†^*P* < 0.05 vs. H/R + F_2_.

Addition of XO/HX (1 mU/ml, 1.2 × 10^−4^ M), EGF (50 ng/ml), or ANISO (40 ng/ml) to F_2_-pretreated H/R CMECs in hypoxia solution for 3 h, followed by culturing in normal medium with F_2_ alone, showed that XO/HX antagonized the effects of F_2_ on H/R-induced ROS generation, MAPK activation, and Egr-1 expression (Figures 8A–E). Similarly, the ERK1/2 and JNK/p38 activators EGF and ANISO, respectively, also antagonized the effects of F_2_ on H/R-induced MAPK activation and Egr-1 expression (Figures 8B–E). These findings indicate that F_2_ can modulate H/R-mediated activation of the ROS/MAPK/Egr-1 signaling pathway in CMECs.

### F_2_ alleviates H/R injury through inhibiting the ROS/MAPK/Egr-1 pathway in CMECs

**Figure 9:**
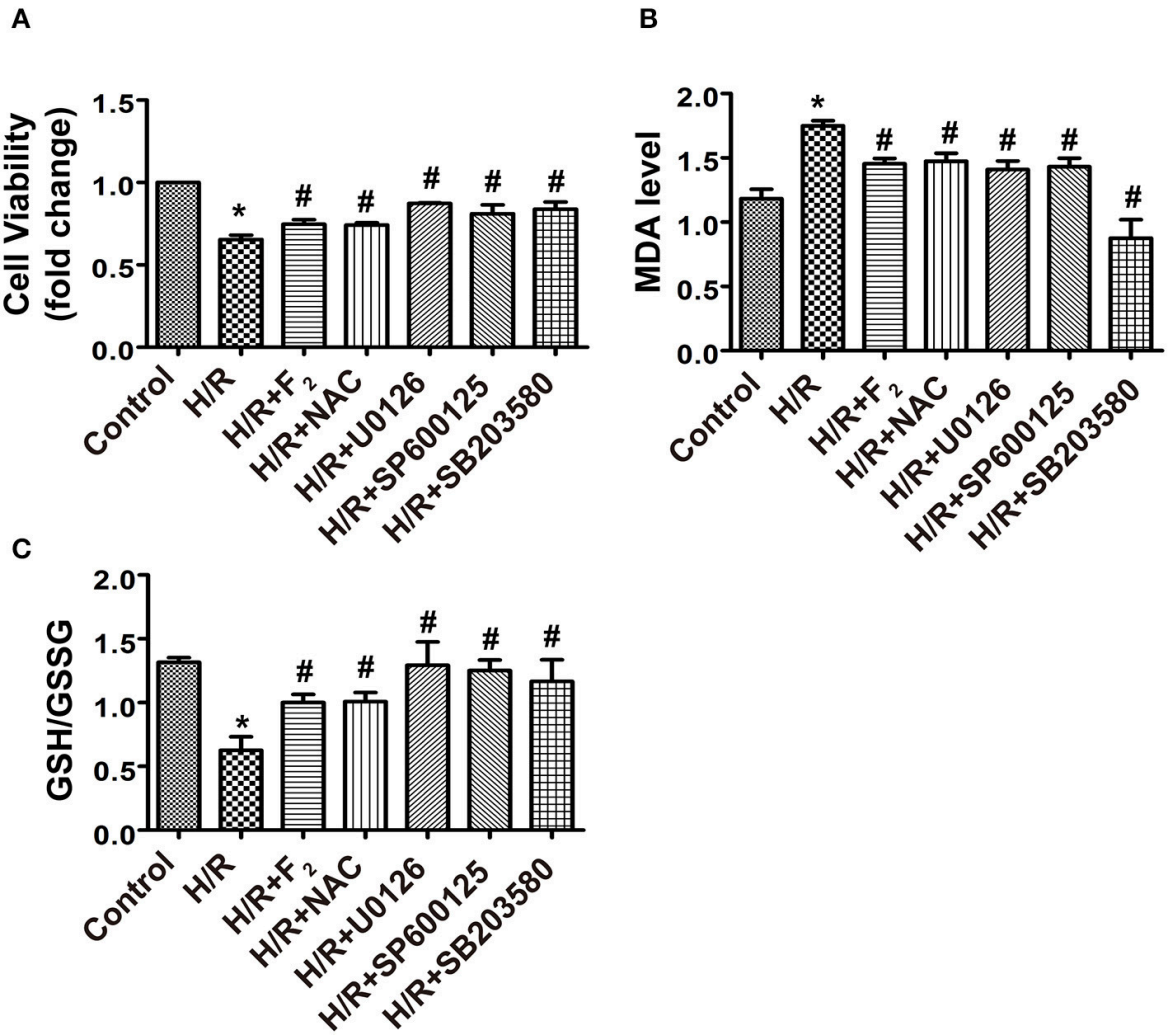
**Effects of signaling pathway inhibitors (NAC, U0126, SP600125, and SB203580) and F_**2**_ on cell viability, MDA levels and the GSH/GSSG ratio of CMECs after H/R. (A)** Cell viability; *n* = 4. **(B)** Level of MDA; *n* = 6. **(C)** GSH/GSSG ratio; *n* = 5. All values are expressed as means ± S.E.M. ^*^P < 0.05 vs. control; ^#^*P* < 0.05 vs. H/R.

F_2_ (10^−6^ M) pretreatment reduced H/R-mediated cell death, as did signaling pathway inhibitors (NAC, U0126, SP600125, and SB203580) (Figure 9A). Since cellular MDA levels and the GSH/GSSG ratio are widely used to reflect the ability to eliminate ROS and the degree of oxidative stress injury, we measured MDA levels and the GSH/GSSG ratio in order to characterize the extent of oxidative damage induced by ROS-mediated signaling. F_2_ (10^−6^ M) reduced MDA levels, but enhanced the GSH/GSSG ratio, compared with H/R alone. Signaling pathway inhibitors exerted the same effects (Figures 9B,C). Induction of Egr-1 is also regarded as a marker of H/R injury (Yan et al., 2000; Zhang et al., 2007, 2008). We found that Egr-1 (red) was expressed at low levels in the control group, and was distributed throughout the entire cell (cytoplasm and nucleus; Supplementary Image 4). Notably, H/R stimulation triggered Egr-1 translocation from the cytoplasm to nucleus. However, this translocation was blocked when F_2_ was present. As expected, NAC and MAPK inhibitors exerted similar effects as F_2_ (Supplementary Image 4). These novel findings indicate that F_2_, similar to inhibitors of ROS and MAPKs, could enhance cell viability, and decrease both oxidative stress and activation of Egr-1 to protect CMECs against H/R injury through inhibiting the ROS/MAPK/Egr-1 pathway.

## Discussion

I/R-related heart diseases are major causes of heart failure and mortality, and acquiring the ability to reduce cardiac I/R injury has been a topic of intense investigation. A growing body of evidence shows that CMECs play a unique and critical role in myocardial I/R progression (Anversa et al., 1980; Brutsaert, 2003; Rohrbach et al., 2015). It is crucial to maintain its integrity and function of the microvascular endothelium in order to reduce myocardial I/R injury. Recent studies by our laboratory suggest that oxidative stress (induced by excessive ROS) and Egr-1 expression are key factors responsible for H/R damage in CMECs. Furthermore, ROS and Egr-1, might crosstalk to mediate H/R injury of CMECs (Zhou et al., 2010a,b; Zhang et al., 2015). Demonstration that an activated ROS/Egr-1 pathway is behind the mechanism of H/R injury in CMECs would be another important step for prevention and therapy of myocardial I/R injury.

In the current study, we show that H/R stimulates rapid generation of ROS and expression of Egr-1 in CMECs. The magnitude of Egr-1 expression corresponds to H/R-induced ROS levels (initiated at H1/R1 and reaching a maximum at H3/R1) in CMECs. Second, application of different doses of the ROS activators XO/HX and scavengers EDA and NAC, to CMECs, results in a dose-dependent stimulatory (XO/XH) or inhibitory (EDA, NAC) effect on ROS levels and Egr-1 expression, indicating ROS levels positively modulate Egr-1 expression. These results support the hypothesis that ROS/Egr-1 signaling occurs in CMECs during H/R, similar to our previous findings using rat H9c2 cardiomyoblast cells.

Early studies on H9c2 cells revealed a JNK-dependent ROS/Egr-1 pathway in H/R injury through initiation of a phosphorylation cascade by ROS, resulting in induction of Egr-1 (Zhang et al., 2015). Previous studies on cardiomyocytes revealed that ERK1/2 also mediates Egr-1-dependent H/R injury (Zhang et al., 2013). Although some studies proposed that JNK and ERK1/2 are the chief upstream signals of Egr-1-mediated lung I/R injury or pulmonary alveolar macrophage H/R injury (Fujita et al., 2004; Yamamoto et al., 2011), p38 also has been shown to contribute to H/R injury in human umbilical vein endothelial cells (Millar et al., 2007). In the present study, activators and inhibitors of ROS and MAPKs were used to determine whether MAPKs mediate ROS/Egr-1 signaling to further contribute to H/R injury in CMECs. The results show that ROS enhance p-ERK1/2, p-JNK, p-p38 and Egr-1 expression, and ROS scavenging inhibits H/R-induced MAPK activation and Egr-1 expression. Therefore, ERK1/2, JNK, p38, and Egr-1 are all downstream signaling molecules of ROS. ERK1/2, JNK, and p38 inhibitors downregulate H/R-induced Egr-1 expression to varying degrees, whereas MAPK activators have the opposite effect on Egr-1 expression, suggesting that ERK1/2, JNK and p38 all act upstream to induce Egr-1. In addition to previous studies on H9c2 cells and cardiomyocytes, the current study further demonstrates that ERK1/2, JNK and p38 all regulate the ROS/Egr-1 signaling pathway in CMECs following H/R.

We also observed that H/R leads to increased MDA levels and a decreased GSH/GSSG ratio in CMECs, suggesting oxidative stress damage occurs during H/R. In addition, H/R-induced oxidative stress damage can be reduced by ROS scavenging and MAPK inhibition. Our previous results showed that an Egr-1 antisense oligodeoxyribonucleotide can antagonize oxidative stress damage in CMECs caused by H/R, as evidenced by decreases in MDA levels and an increase in SOD activity (Zhou et al., 2010a,b). Based on this, we deduce that oxidative stress-related damage in CMEC H/R injury, triggered by abnormal ROS generation, is mediated by the activated ROS/MAPK/Egr-1 pathway.

We also previously showed that F_2_ protects myocardium against I/R injury by blocking L-type calcium channels, inhibiting Egr-1 gene and protein expression, and reducing damage due to oxidative stress. Although we have known that F_2_ reduces H/R-induced oxidative stress injury and other damage in CMECs through inhibiting expression of Egr-1, the cellular target of F_2_ remains unknown. Studies on H/R H9c2 cells demonstrate that F_2_ inhibits the ROS/JNK/Egr-1 pathway (Zhang et al., 2015). Data from the present study show that F_2_ reduces H/R-induced increases in ROS level, MAPK activation, and Egr-1 expression in CMECs in a dose-dependent manner. In addition, we observe that F_2_ has multiple effects on Egr-1: F_2_ not only inhibits Egr-1 mRNA and Egr-1 protein expression, but also reduces Egr-1 nuclear translocation. Moreover, we show, by EMSA, that F_2_ downregulates Egr-1 activity and that ROS donor administration antagonizes the F_2_-mediated inhibition of ROS induction, MAPK activation and enhanced Egr-1 expression, which are induced by H/R stimuli. Similarly, EGF, an ERK1/2 activator, and ANISO, a JNK and p38 activator, inhibit F_2_-mediated decreases in MAPK activation and Egr-1 expression, suggesting that F_2_ reduces Egr-1 expression by inhibiting ROS generation and MAPK activation in CMECs.

Moreover, in H/R CMECs, F_2_, as well as inhibitors of ROS and MAPKs, increase cell viability, reduce oxidative stress injury, as demonstrated by a decreased MDA level and an increased GSH/GSSG ratio, and inhibition of nuclear translocation of Egr-1, all of which have been shown to be associated with I/R-induced inflammation and other types of injury (Yan et al., 2000; Zhang et al., 2007, 2008; Zhou et al., 2010a). Taken together, our data suggest F_2_ protects CMECs via regulating the ROS/MAPK/Egr-1 signaling pathway during H/R. In addition, this study provides a necessary and sufficient explanation for Egr-1's central and unifying role in the pathogenesis of I/R injury, i.e., Egr-1 also mediates ROS-induced oxidative stress injury caused by I/R. Our findings provide guidance for I/R-related investigations of other organs in which the microvascular endothelium is indispensable. In addition to I/R, ROS induced oxidative stress turn out to be primary pathogenesis of other diseases such as diabetes, heart failure and Alzheimer's diseases, therefore, it is reasonable to expect that ROS in above diseases might also active ROS/MAPK/Egr-1 pathway, and even damage vascular endothelium, which can be ameliorated similarly by F_2_.

## Conclusions

In summary, H/R leads to ROS/Egr-1 signaling pathway activation in CMECs, and MAPK activation mediates the signaling pathways between ROS and Egr-1. F_2_ downregulates H/R-induced ROS/MAPK/Egr-1 signaling to antagonize myocardial I/R injury.

## Author contributions

GS and FZ supervised the overall project and helped writing the paper; SL performed the experiment and drafted the manuscript; YZ designed the experiment and analyzed the data; SZ polished the English to improve the quality of this manuscript; FG, YC, and WL contributed to figure handing or the statistical analysis.

### Conflict of interest statement

The authors declare that the research was conducted in the absence of any commercial or financial relationships that could be construed as a potential conflict of interest.

## Supplementary material

Supplementary Image 1 **Letter of patent of F_**2**_**.

Supplementary Image 2 **Chemical structure of (A)** Hal and **(B)** F_2._

Supplementary Image 3 **Identification of microvascular endothelial cells (CMECs) by immunofluorescence analysis of CD31. CD31 (green), Hoechst 33258 (blue) (400 ×, bar = 20 μm)**.

Supplementary Image 4 **Effects of signaling pathway inhibitors and F_**2**_ on nuclear translocation of Egr-1 in CMECs after H/R, as assessed using immunofluorescence**. Egr-1 in the control group (light red, low level Egr-1) is mainly expressed in cytoplasm. In the H/R group, Egr-1 (bright red) was observed in the nucleus and red fluorescence was nearly undetectable in the cytoplasm, indicating Egr-1 nuclear translocation. When pre-treated with F_2_, NAC, U0126, SP600125, and SB203580, cells showed decreased Egr-1 expression and translocation to the nucleus.
