# Supplementary figures and images for: N-n-butyl Haloperidol Iodide Protects against Hypoxia/Reoxygenation Injury in Cardiac Microvascular Endothelial Cells by Regulating the ROS/MAPK/Egr-1 Pathway
Source: Front Pharmacol. 2017 Jan 5;7:520. doi: 10.3389/fphar.2016.00520 (PMC5216659; doi:10.3389/fphar.2016.00520)

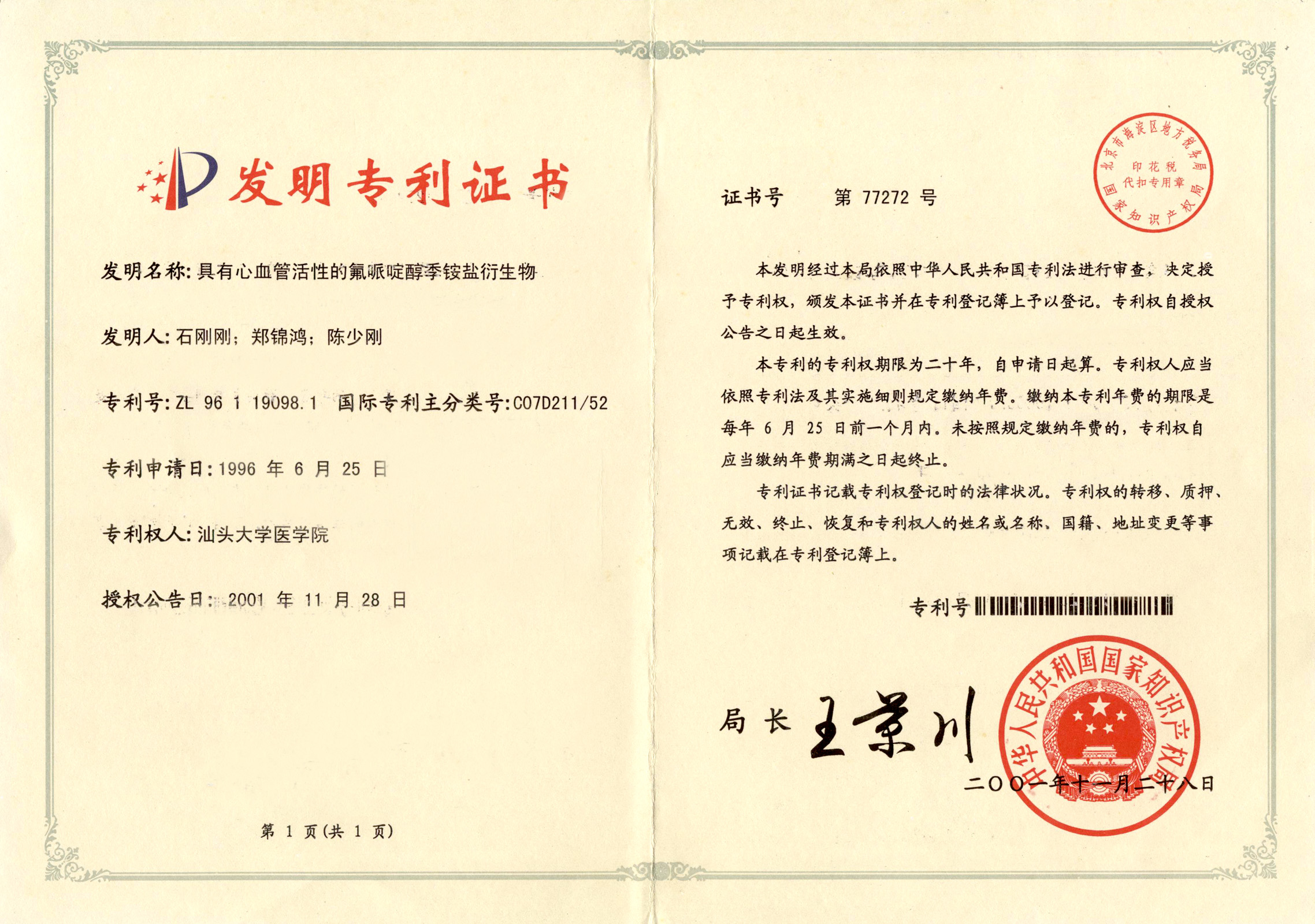

Supplement: Supplementary Image 1 — Letter of patent of F2. [file Image1.JPEG]

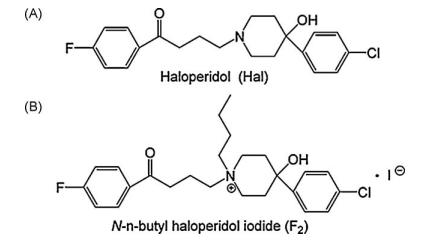

Supplement: Supplementary Image 2 — Chemical structure of (A) Hal and (B) F2. [file Image2.JPEG]

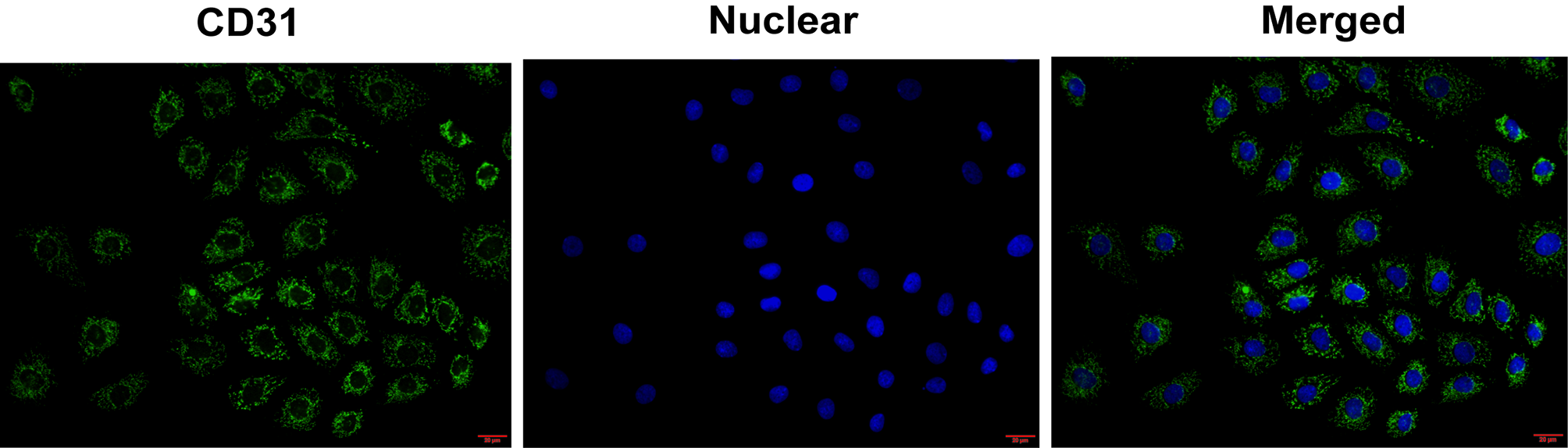

Supplement: Supplementary Image 3 — Identification of microvascular endothelial cells (CMECs) by immunofluorescence analysis of CD31. CD31 (green), Hoechst 33258 (blue) (400 ×, bar = 20 μm). [file Image3.TIF]

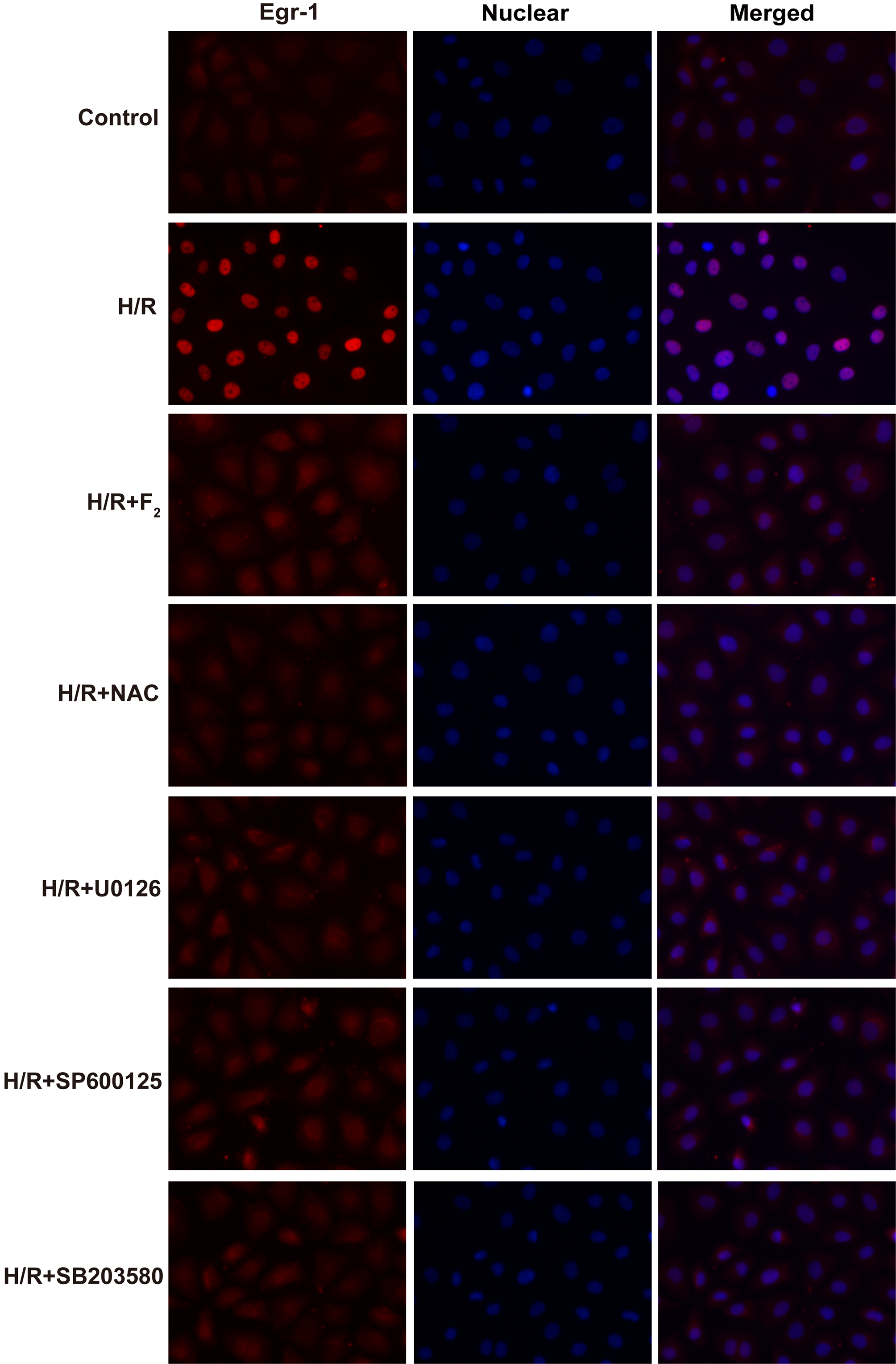

Supplement: Supplementary Image 4 — Effects of signaling pathway inhibitors and F2 on nuclear translocation of Egr-1 in CMECs after H/R, as assessed using immunofluorescence. Egr-1 in the control group (light red, low level Egr-1) is mainly expressed in cytoplasm. In the H/R group, Egr-1 (bright red) was observed in the nucleus and red fluorescence was nearly undetectable in the cytoplasm, indicating Egr-1 nuclear translocation. When pre-treated with F2, NAC, U0126, SP600125, and SB203580, cells showed decreased Egr-1 expression and translocation to the nucleus. [file Image4.TIF]
